# Short-Term Efficacy of Ultramicronized Palmitoylethanolamide in Peripheral Neuropathic Pain

**DOI:** 10.1155/2014/854560

**Published:** 2014-05-20

**Authors:** Dario Cocito, Erdita Peci, Palma Ciaramitaro, Aristide Merola, Leonardo Lopiano

**Affiliations:** Department of Neuroscience, AOU Città della Salute e della Scienza di Torino, University of Turin, Via Cherasco 15, 10124 Torino, Italy

## Abstract

*Introduction*. This study evaluates the efficacy of palmitoylethanolamide ultramicronized (PEA-um) as an add-on treatment in patients with diabetic or traumatic neuropathic pain (NP). *Methods*. 30 patients with chronic NP were assessed with Visual Analogue Scale (VAS), NP Symptom Inventory (NPSI), and Health Questionnaire Five Dimensions (EQ-5D), both at baseline and after 10 and 40 days of treatment with 1200 mg/die of PEA-um. All other therapies were maintained stable during the follow-up period. *Results*. VAS mean score significantly improved within the first 10 days, ranging from 8.20 ± 1.53 to 6.40 ± 1.83 (*P* < 0.002), with a further decrease to 5.80 ± 2.04 (*P* < 0.001) after 40 days of PEA-um administration. Moreover, NPSI total score improved from 5.2 ± 1.5 to 3.8 ± 2.1 (*P*: 0.025) and EQ-5D ranged from −0.30 ± 0.65 to 0.5 ± 0.34 (*P* < 0.001) between T0 and T2. *Conclusions*. This study reports the prospective short-term efficacy data of oral PEA-um in patients with diabetic or traumatic NP. A significant improvement was observed both in VAS and NPSI scores and in quality of life scales after 40 days of treatment, although some limitations should be considered, including the short followup and the open-label study design.

## 1. Introduction

Neuropathic pain (NP) due to nerve injury or neuronal dysfunction could be associated with inflammatory reactions and mobilization of the immune system cells [1]. Microglia are thought to coordinate the inflammatory responses of the central nervous system (CNS), while mast cells have a pivotal role in the inflammatory responses of the peripheral nervous system (PNS). Palmitoylethanolamide (PEA-um) is an endogenous fatty acid, which can inhibit the release of proinflammatory mediators from activated mast cells, reducing the recruitment and activation of mast cells at sites of nerve injury [2]. PEA-um demonstrated a significant efficacy on pain in the murine model of diabetic neuropathy [3]. However, few data are currently available in human subjects; sporadic cases have been reported in the literature [4–8], suggesting that PEA-um oral administration may lead to an improvement of NP, reducing the allodynia and hyperalgesia.

In this context our aim was to evaluate the clinical efficacy of oral PEA-um treatment in a cohort of patients with diabetic or traumatic chronic NP, not adequately controlled by other oral conventional therapies. Subjects were included in an open label study, evaluating the efficacy of oral PEA-um administration, as an add-on therapy, after 10 and 40 days of therapy.

## 2. Materials and Methods

A cohort of 30 patients with diabetic or traumatic chronic NP, not controlled by other oral conventional therapies, was included in this study. All subjects suffered from a chronic pain for at least 100 days at the time of recruitment, with a Visual Analogue Scale (VAS) pain score higher than 6 [9], in spite of the best therapeutic regimen with Pregabalin, Gabapentin, and/or Tramadol. All patients included in the study were diagnosed as having an NP, according to the DN4 score ≥4, with an average score of 6.60 (range 4–9), and a stable intensity of pain in the preceding 100 days.

After a preliminary screening, patients were asked to participate at the study and then were evaluated by means of VAS scale, NP Symptom Inventory (NPSI) [10], and health questionnaire five dimensions (EQ-5D) for quality of life [11]. NPSI single items were also evaluated separately: burning (superficial) spontaneous pain (Factor 1); pressing (deep) spontaneous pain (Factor 2); paroxysmal pain (Factor 3); evoked pain (Factor 4); paresthesia/dysesthesia (Factor 5). This study was authorized by the scientific board of our institute and all subjects signed a written informed consent.

Oral PEA-um treatment was initiated at the doses of 1200 mg/die in sachet formulation for the first 10 days and 1200 mg/die in tablet formulation between the 10th and 40th days. The dosages of all other therapies were maintained stable during the entire duration of the study.

After the first evaluation, performed at the time of inclusion (T0), patients were reassessed at fixed intervals: VAS evaluation was performed in all subjects after 10 days of treatment (T1), while VAS, NPSI, EQ-5D, and DN4 were repeated after 40 days of treatment (T2).

The statistical comparisons at different time points (T0, T1, T2) were performed by means of Friedmann rank sum test and where appropriate using Wilcoxon rank sum test. A repeated measure ANOVA was performed in order to evaluate whether patients responders to PEA-um had a better outcome in the NPSI and EQ-5D scores. The analyses were performed using PASWStat 18 for Windows. Data are reported as mean values ± standard deviations and all *P* values are two-tailed, considering 0.05 as the statistical threshold.

## 3. Results

The main clinical and demographic characteristics of patients at baseline are reported in Table 1. During the study three subjects dropped out for reasons not related to PEA-um administration (one patient underwent surgery for colecistitis, one patient had a vertebral fracture, requiring hospitalization, and one patient had an intestinal virus); clinical data were then available for 27 patients (14 males and 13 females); 20/27 subjects suffered from diabetic neuropathic pain, and 7/27 subjects were affected by neuropatic pain consequent to brachial plexus traumatic lesions.

At T0 evaluation VAS mean score was 8.20 ± 1.53 (range 6–10), NPSI total score was 5.2 ± 1.5 (range 2. 4–8.7), and EQ-5D mean score was −0.30 ± 0.65 (range from −1.85 to 0.65).

At the first evaluation (T1) the VAS mean score decreased from 8.20 ± 1.53 to 6.4 ± 1.83 (range 2–10), reaching the statistical threshold (*P* value < 0.002), and this improvement was even more evident at the T2 evaluation (*P* value < 0.001), with a further decrease in the mean VAS score to 5.80 ± 2.04 (range 2–10).

Moreover, as shown in Table 2, a significant improvement was observed also in the NPSI total score, which decreased from the T0 values of 5.2 ± 1.5 to the T2 values of 3.8 ± 2.1 (*P*: 0.025), and a similar trend was observed for the EQ-5D mean score, which increased from the T0 value of −0.30 ± 0.65 to the T2 value of 0.50 ± 0.34 (*P* < 0.001). According to the repeated measure ANOVA analysis, patients showing a VAS scale improvement had a significantly better outcomes at the NPSI (*P*: 0.007) and at the EQ-5D (*P* < 0.001) scores.

A separate analysis of the NPSI different section scores (Table 2) revealed that the most evident improvement was achieved in section 5 of the scale, which refers to paresthesia and dysesthesia (*P*: 0.003).

## 4. Discussion

Brachial plexus neuropathy, with or without spinal cord root avulsion, and diabetic neuropathy are both frequent causes of NP, with a prevalence that may vary depending on definitions, populations, and methodologies used for the assessment of NP. Large international multicenter cohort studies [12, 13] report a 10–30% prevalence of NP in diabetic patients, while an Italian study described a 69% incidence of NP in 55 patients with traumatic plexopathies [14].

It has been reported that NP severity can affect the patient's quality of life more than neuropathic disability and clinical severity [15], suggesting that its symptomatic management might be one of the most important targets in the treatment of peripheral neuropathies. However, in spite of the relatively high prevalence, little consensus exists on the best primary therapeutic choices in NP, also in view of the multiple complexities underlying etiopathogenic factors.

PEA demonstrated a significant efficacy in reducing the hyperalgesic component of NP [3, 16, 17], modulating the activity of mast cells. Moreover, PEA might help to restore the peripheral nerve sensitization process, induced by the release of inflammatory cytokines at the site of nerve injury, through a modulation of the endoneurial mast cells hyperactivity [3]. However, the highly lipophilic PEA crystalline structure has a poor oral adsorption, thus requiring to be micronized and converted into particles with an elevated surface area to volume ratio, in order to enhance its assimilation [18].

In this study, we report the first prospective short-term data of oral PEA-um treatment in patients with diabetic or traumatic NP. All subjects included in this protocol had been already treated with Pregabalin, Gabapentin, or Tramadol, with only partial improvement of symptoms and a significant residual pain, as demonstrated by the VAS score higher than 6 at the time of recruitment.

Over the 40 days observational follow-up period, we observed a significant improvement of VAS score, with an initial reduction after 10 days of therapy and a further decrease at the end of the study. In addition, also the NPSI score related to paresthesia and dysesthesia significantly improved, as well as the quality of life measured with the EQ-5D scale.

Overall, our findings would suggest that PEA-um oral administration might be effective as an add-on therapy in the control of NP, also resulting in an amelioration of patient quality of life. However, two limitations should be considered in the interpretations of our data: this study reports the short-term follow-up data of a single center open-label study, and the absence of a comparative group does not allow speculations on the possible placebo effect in relieving pain. Further larger prospective studies with longer follow-up duration seem therefore necessary to confirm our findings and the long-term possible efficacy of oral PEA-um treatment in the management of peripheral neuropathic pain.

## Figures and Tables

**Table 1:** Clinical and demographic characteristics of patients at baseline.

	Diabetic neuropathy	Traumatic neuropathy
Number of subjects	23	7
Gender	12 males	4 males
	11 females	3 females
Age (years old)	63.87 ± 13.4	57.14 ± 7.5
VAS score	7.98 ± 1.7	8.57 ± 1.4
NPSI score	5.4 ± 1.6	4.9 ± 2.1
EQ-5D score	−0.12 ± 0.35	−0.78 ± 1.03

Main clinical and demographic characteristics of patients affected by diabetic neuropathy and traumatic neuropathy at baseline. VAS: Visual Analogue Scale; NPSI: Neuropathic Pain Symptom Inventory; EQ-5D: health questionnaire five dimensions.

**Table 2:** Baseline to follow-up scores.

	Baseline	Followup	*P* value
VAS score	8.20 ± 1.53	5.80 ± 2.04	*P* < 0.001*
NPSI total score	5.2 ± 1.5	3.8 ± 2.1	*P*: 0.025*
(i) Burning	7.2 ± 2.7	5.6 ± 3.2	*P*: 0.063
(ii) Pressing	5.4 ± 2.9	4.0 ± 3.0	*P*: 0.073
(iii) Paroxysmal pain	5.6 ± 3.1	4.0 ± 3.0	*P*: 0.061
(iv) Evoked pain	3.7 ± 2.3	3.0 ± 2.2	*P*: 0.279
(v) Paresthesia/dysesthesia	5.9 ± 2.6	3.6 ± 2.9	*P*: 0.003*
EQ-5D score	−0.30 ± 0.65	0.50 ± 0.34	*P* < 0.001*

A significant improvement was observed in the VAS and NPSI scores between baseline and followup. Moreover, the NPSI subscores showed a highly significant improvement for the paresthesia/dysesthesia subscore and a trend towards amelioration for the other subscores.

VAS: Visual Analogue Scale; NPSI: Neuropathic Pain Symptom Inventory; EQ-5D: health questionnaire five dimensions.

*Indicates the statistical significance (*P* value < 0.05).
